# Supplementary material for: The microbiome biomarkers of pregnant women’s vaginal area predict preterm prelabor rupture in Western China
Source: Front Cell Infect Microbiol. 2024 Oct 31;14:1471027. doi: 10.3389/fcimb.2024.1471027 (PMC11560878; doi:10.3389/fcimb.2024.1471027)
Supplement: Supplementary file 1 [file DataSheet1.zip › compare_1/Community/Pieplot/class/sample/P22.class.pieplot.pdf]

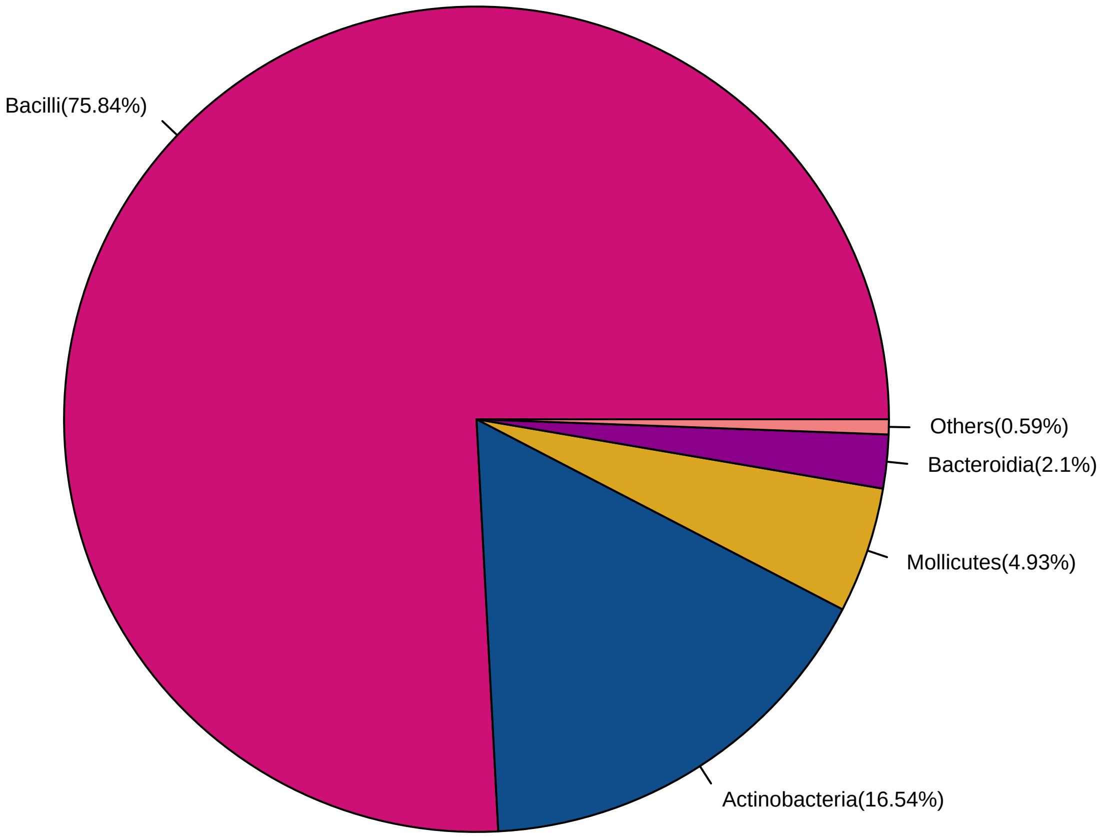
Bacilli(75.84%)
Others(0.59%)
Bacteroidia(2.1%)
Mollicutes(4.93%)
Actinobacteria(16.54%)
